# Development of a full-process non-pharmacological labor pain management protocol for vaginal delivery: a Delphi study

**DOI:** 10.3389/fmed.2026.1849246

**Published:** 2026-10-07

**Authors:** Xiaoxing Xie, Rongrong Wu, Jianwen Zhang, Yan Shen, Xinqun Li

**Affiliations:** 1 Delivery Room, Women and Children's Hospital of Ningbo University, Ningbo, Zhejiang, China; 2 Postpartum Maternal and Child Health Centers, Women and Children's Hospital of Ningbo University, Ningbo, Zhejiang, China

**Keywords:** Delphi study, labor pain management, non-pharmacological labor analgesia, protocol development, vaginal delivery

## Abstract

**Background:**

Non-pharmacological strategies can complement pharmacological labor analgesia, yet their clinical use is often fragmented across techniques and labor stages, limiting consistent and continuous support during vaginal delivery.

**Objective:**

To develop a consensus-based, implementation-oriented clinical practice protocol that organizes full-process non-pharmacological labor pain management from prenatal preparation through early postpartum evaluation.

**Methods:**

Guided by the Manchester pain management model and CREDES recommendations, this Delphi-based protocol development study generated an initial framework through structured evidence review, focus group discussion, and expert consultation. Eleven interdisciplinary experts completed two anonymous Delphi rounds. Group-level statistical feedback and anonymized comments from round 1 informed the round-2 questionnaire, and predefined consensus criteria guided protocol finalization.

**Results:**

The response rate was 100% in both rounds, and the expert authority coefficient was 0.92. First-round coefficients of variation ranged from 0.00 to 0.28; Kendall's W values were 0.230, 0.215, and 0.208 for primary, secondary, and tertiary items, respectively (*P* = 0.021, 0.001, and < 0.001). Second-round coefficients of variation ranged from 0.00 to 0.19; cold therapy (D4-4; CV = 0.19) was retained as a conditional item requiring individualized assessment. Kendall's W values were 0.240, 0.225, and 0.212, respectively (*P* = 0.010, 0.001, and < 0.001). The final protocol comprised five primary domains, 20 secondary items, and 57 tertiary items integrating pain-management objectives, team building, dynamic assessment, mechanism-organized interventions, shared decision-making, and outcome evaluation from prenatal preparation to 24 h postpartum.

**Conclusion:**

This study shows that dispersed evidence on non-pharmacological labor pain management can be translated through multidisciplinary consensus into a coherent full-process clinical pathway. The protocol offers a standardized framework to complement pharmacological analgesia and support consistent, individualized care; its feasibility, acceptability, and clinical effects require prospective multicenter evaluation.

## 1 Introduction

Labor pain is one of the most prominent symptoms experienced by women during vaginal delivery. When severe and insufficiently relieved, it can contribute to maternal stress responses, fear of childbirth, negative birth experience, and difficulties with maternal cooperation during labor. These effects may interact with labor progress and maternal-neonatal outcomes, although the magnitude of risk varies across populations and care settings (1, 2). Therefore, labor pain management should address both analgesic effectiveness and women's psychological, emotional, and informational needs.

Labor analgesia generally includes pharmacological and non-pharmacological methods. Contemporary pharmacological analgesia, especially neuraxial labor analgesia, is an effective and widely used option; its influence on labor duration or instrumental delivery varies by drug concentration, timing, obstetric context, and contemporary anesthetic technique (1, 3, 4). Non-pharmacological approaches do not replace pharmacological analgesia. Instead, they can be applied before, during, and after intrapartum analgesic decision-making as low-risk adjunctive measures that support coping, comfort, maternal participation, and shared decision-making (1, 2).

However, the implementation of non-pharmacological labor analgesia remains inconsistent, and standardized procedures are not yet routinely embedded across institutions or levels of care. Existing guidelines and evidence summaries recommend multiple non-pharmacological approaches, but clinical application often depends on local resources, staff training, and institutional workflow (5–8). Moreover, many studies focus on single techniques, such as birthing balls, breathing techniques, massage, music therapy, acupressure, or TENS, or on specific labor stages, with limited attention to continuous management spanning prenatal preparation, intrapartum support, and early postpartum evaluation (6–8).

The four stages of the Manchester pain management model correspond closely to the sequential phases of labor. The preparation stage involves establishing a multidisciplinary pain management team and providing professional education for healthcare personnel. The assessment stage extends throughout the entire labor process, including prenatal pain perception assessment and intrapartum pain monitoring. The intervention stage covers intrapartum non-pharmacological pain relief measures alongside maternal and family health education. The recovery stage continues through the first 24 h postpartum, focusing on evaluation of pain management outcomes (9, 10). Compared with single-intervention models that emphasize only intrapartum pain relief, this framework offers a more systematic and integrated approach. For instance, although Gallo et al. proposed a sequential intervention protocol spanning multiple labor stages, its emphasis was primarily on physical intrapartum interventions, whereas the present protocol also incorporates prenatal preparation, team structure, assessment, shared decision-making, and early postpartum evaluation (6).

At present, there remains a substantial research gap in full-process non-pharmacological labor pain management for vaginal delivery both domestically and internationally. Although some international frameworks integrate multiple interventions, locally adaptable implementation details for Chinese obstetric settings remain limited (5). Conversely, domestic studies are commonly confined to individual labor stages or combined use of selected techniques, and a comprehensive system integrating objectives, team structure, assessment, interventions, shared decision-making, and outcome evaluation has not yet been fully established (7, 8, 11–13). Therefore, grounded in the Manchester pain management model, this study aimed to develop a consensus-based, implementation-oriented clinical practice protocol for full-process non-pharmacological labor pain management among women undergoing vaginal delivery. The novelty of the study lies not in generating new efficacy evidence for individual non-pharmacological interventions, but in organizing existing evidence and expert consensus into a structured, full-process protocol for future implementation and validation. Accordingly, the study addresses an implementation gap by transforming heterogeneous evidence into an operational pathway rather than evaluating the efficacy of any single intervention.

## 2 Methods

This study was designed as a Delphi-based clinical protocol development study rather than a systematic review. The literature search was used to generate the initial item pool and was followed by focus group discussion and two rounds of anonymous expert consultation.

### 2.1 Development of the preliminary draft of the non-pharmacological pain management protocol for vaginal delivery

#### 2.1.1 Literature search strategy

Chinese databases, including China National Knowledge Infrastructure, Wanfang Data, VIP, and SinoMed, were searched using the keywords “阴道分娩/自然分娩/阴道试产 (vaginal delivery/natural childbirth/trial of labor)” and “分娩疼痛/非药物性分娩镇痛/疼痛管理 (labor pain/non-pharmacological labor analgesia/pain management).” English databases, including PubMed, Embase, UpToDate, JBI, and the Cochrane Library, were searched using the terms “vaginal birth/vaginal delivery/natural childbirth/spontaneous vaginal delivery/trial of labor/labor pain/childbirth pain/delivery pain/non-pharmacological pain relief/non-pharmacologic interventions/non-drug analgesia/non-medicated birth/pain management.” Clinical practice guideline repositories included the World Health Organization, National Institute for Health and Care Excellence, Scottish Intercollegiate Guidelines Network, National Guideline Clearinghouse, Canadian Medical Association Infobase, International Guideline Collaboration Network, and Medlive. The search covered all publications from database inception through December 2024.

#### 2.1.2 Inclusion and exclusion criteria

Inclusion criteria were as follows: (1) Study participants were women undergoing vaginal delivery; (2) The content involved non-pharmacological labor analgesia measures, pain management processes, or full-process intervention models; (3) Study type was a guideline, systematic review, clinical decision-making document, or evidence summary; (4) Language was Chinese or English; (5) No restriction on publication date (as earlier seminal works may provide theoretical underpinnings).

Exclusion criteria were as follows: (1) Studies focusing exclusively on pharmacological analgesia; (2) Studies targeting specific maternal conditions (*e.g.*, hypertensive disorders of pregnancy); (3) Case reports, abstract-only reviews, or conference abstracts; (4) Duplicated publications or incomplete data.

Based on these criteria, a total of nine documents were included: three clinical guidelines (4, 5, 10), two clinical decision-making frameworks (14, 15), one systematic review (16), and three evidence summaries (7, 17, 18).

#### 2.1.3 Literature quality appraisal and evidence-to-item transformation

The *Appraisal of Guidelines for Research and Evaluation II* was used to assess the methodological quality of the three included guidelines across six domains: scope and purpose, stakeholder involvement, rigor of development, clarity of presentation, applicability, and editorial independence. All three guidelines achieved a rating of Grade B or above (≥60 points), confirming the reliability of the evidence base. The research team employed a “evidence extraction–clinical matching–item generation” three-step approach to translate the evidence into operational components of the protocol: (1) Core elements were systematically extracted from the included literature, such as non-pharmacological analgesic techniques, assessment tools, and team composition. (2) To ensure practical relevance, the extracted evidence was reviewed by a multidisciplinary expert panel consisting of obstetricians, senior midwives, and pain management nurses. The evidence was screened across three dimensions, namely labor-stage adaptability, operational feasibility, and maternal-neonatal safety. Interventions limited to a single stage of labor (e.g., those applicable only to the active phase of the first stage) were excluded, while continuous measures were retained from prenatal preparation through 24 h postpartum. Techniques requiring specialized equipment or advanced technology that are impractical in primary care settings were removed, giving priority to simple, scalable, and easy-to-implement interventions. Safety compliance with obstetric standards was verified to ensure alignment with real-world clinical practices, such as proper temperature regulation for hot compress therapy and contraindication checks for transcutaneous electrical nerve stimulation (TENS). (3) The validated evidence was converted into operational items. For instance, “continuous pain assessment” was refined into specific evaluation nodes, such as during labor onset, at cervical dilation of 2.5 cm, and at full dilation. Through this process, a preliminary framework comprising 5 primary, 23 secondary, and 68 tertiary items was established.

### 2.2 Expert consultation using the Delphi method

#### 2.2.1 Design of the consultation questionnaire

An expert consultation questionnaire consisting of four main components was developed using the Delphi method: (1) Cover letter to experts: An overview of the study's background, objectives, and significance was provided. (2) Expert demographic information form: Demographic data were collected, including sex, age, educational background, professional title, years of work experience, and research field. (3) Expert authority self-assessment form: Each expert's familiarity with the research topic and the basis of their professional judgment were assessed. (4) Expert evaluation form for the proposed full-process non-pharmacological pain management protocol for vaginal delivery: Each item was rated using a five-point Likert scale (1 = not important at all, 5 = extremely important) to evaluate its importance. Open-ended sections were provided for experts to offer modification suggestions, propose deletions, or recommend additional items.

To ensure the scientific rigor of the questionnaire, a pilot test was conducted. Three experts not participating in the formal Delphi rounds (two obstetric nursing specialists and one biostatistics expert) were invited to evaluate the content validity of the questionnaire. The content validity index was calculated as 0.92, indicating strong content validity. A test-retest reliability assessment was then performed by administering the questionnaire twice to 10 midwives, 2 weeks apart, yielding a reliability coefficient of 0.86, confirming good reliability and validity. This study was reviewed and approved by the hospital's medical ethics committee (Approval No. EC2024-067).

#### 2.2.2 Selection of Delphi experts

A total of 11 experts from Fujian, Zhejiang, Shandong, and Shanghai were recruited for consultation. The panel size was selected to balance disciplinary diversity, feasibility of repeated rating, and stability of expert participation, consistent with Delphi studies in which credibility depends on expert heterogeneity, independence, and iterative consensus-building rather than statistical sampling (19). Expert selection was conducted through multiple channels to ensure diversity and representativeness. The recruitment process was based on the obstetric specialty collaboration network affiliated with the research institution, supplemented by recommendations from national professional bodies, such as the Pediatrics and Obstetrics Specialized Committee of the Chinese Nursing Association and the Pain Management Society. In addition, targeted invitations were extended to authors of high-impact publications in relevant fields to ensure both disciplinary breadth and professional authority. Because the protocol was designed as a professional clinical practice and implementation framework, the Delphi panel consisted of clinical and management experts; women with childbirth experience were not included in the formal Delphi panel, and this design choice is addressed in the limitations. Inclusion criteria for experts were as follows: (1) Engaged in fields such as obstetric nursing, nursing management, midwifery, obstetrics, or pain management for ≥10 years; (2) Holding a bachelor's degree or higher; (3) Possessing an intermediate or senior professional title; (4) Providing informed consent and voluntarily participating in the study. As to the exclusion criteria, experts who failed to return questionnaires on time or were unable to continue participation for any reason were excluded from subsequent rounds.

#### 2.2.3 Implementation of the Delphi consultation

From April to May 2025, the consultation questionnaires were distributed to the experts individually via email or one-to-one WeChat messages. No group chat or collective scoring meeting was used. A trained research secretary collected all responses separately, assigned coded identifiers, and removed identifying information before statistical synthesis, so experts did not know each other's scores or comments. The Delphi process was conducted with reference to the CREDES recommendations (19). In the first round, experts rated the importance of each item and provided written feedback. Before the second round, each expert received a controlled feedback package containing group-level mean scores, standard deviations, coefficients of variation, item-level retention proposals, and anonymized representative comments from the first round. Qualitative comments were extracted verbatim, classified by item and theme, and discussed by the research team together with the quantitative indicators. Items were retained, merged, refined, or removed according to the predefined thresholds, clinical feasibility, maternal-neonatal safety, and consistency with the Manchester pain management model. Disagreements within the research team were resolved by consensus after reviewing the statistical indicators and original comments; if consensus could not be reached, the decision was made by majority vote of the core research group. All items requiring clarification or addition were returned to the Delphi panel in the second-round questionnaire for re-rating. Items were removed if their mean importance score was < 4.0 or CV exceeded 0.25. The consultation concluded once expert opinions reached consensus. According to the pre-established stopping criteria, the Delphi process would terminate if Kendall's coefficient of concordance (W) ≥0.20 with *P* < 0.05 and the modification rate of expert comments < 10% after two rounds. Following the second round, the analysis yielded Kendall's W values of 0.240, 0.225, and 0.212 for primary, secondary, and tertiary items, respectively, with P values of 0.010, 0.001, and < 0.001. Four written comments were received in the second round and led to five item-level decisions; the remaining modification rate was below 10%. As the consensus threshold was achieved, the Delphi process was formally concluded.

### 2.3 Statistical analysis

All data were entered and analyzed using Excel 2021 and SPSS 26.0. Categorical variables were expressed as frequency and percentage (*n*[%]), while continuous variables were presented as mean ± standard deviation (x ± s). Expert enthusiasm was evaluated by the effective response rate. The expert authority coefficient (Cr) was calculated as Cr = (Ca + Cs)/2, where Ca represents the judgment basis coefficient and Cs the familiarity coefficient; a Cr value >0.70 indicates an acceptable level of authority. Consensus among experts was assessed using the CV and Kendall's W. The CV, calculated as standard deviation divided by mean, reflects the degree of dispersion among item ratings; CV values were calculated from unrounded source data and rounded to two decimals for reporting. Kendall's W was calculated separately for primary, secondary, and tertiary items, with *P* < 0.05 indicating statistical significance.

## 3 Results

### 3.1 Basic information of experts

A total of 11 experts completed both Delphi rounds. Their mean age was 44.45 ± 5.39 years, with an average of 22.64 ± 7.53 years of professional experience. Regarding educational background, four held doctoral degrees, three master's degrees, and four bachelor's degrees. In terms of professional titles, four held senior, five associate senior, and two intermediate titles. As to professional fields, the expert panel encompassed obstetrics (1), nursing management (3), obstetric nursing (2), midwifery (3), and pain management (2). All experts met the inclusion criteria.

### 3.2 Expert enthusiasm and authority

Across both Delphi rounds, 11 questionnaires were distributed and retrieved each time, achieving a 100% effective response rate, indicating a high level of expert enthusiasm. In the first round, 8 experts (72.7%) proposed 18 item-level suggestions; in the second round, four written comments were received and led to five item-level decisions. The Ca value was 0.95, the Cs value was 0.88, and the resulting Cr was 0.92 (>0.70), demonstrating an ideal level of expert authority.

### 3.3 Degree of expert consensus

In the first Delphi round, the CV across all item levels ranged from 0.00 to 0.28, with primary items ≤ 0.13, secondary items ≤ 0.23, and tertiary items ≤ 0.28, indicating low dispersion and initial consistency. The Kendall's W values were 0.230 (*P* = 0.021) for primary items, 0.215 (*P* = 0.001) for secondary items, and 0.208 (*P* < 0.001) for tertiary items, all reaching statistically significant levels of concordance. In the second Delphi round, CV values ranged from 0.00 to 0.19. All items were within 0.00–0.15 except cold therapy (D4-4), which had a CV of 0.19 and was retained as a conditional item requiring individualized assessment and careful application. The Kendall's W values increased to 0.240 (*P* = 0.010), 0.225 (*P* = 0.001), and 0.212 (*P* < 0.001) for primary, secondary, and tertiary items, respectively. These statistically significant W values indicate modest but improving concordance among experts rather than high agreement. The pattern of decreasing CV values and statistically significant Kendall's W values supports acceptable consensus for protocol development. Collectively, the two-round pattern indicates that the protocol items were rated as important with generally low dispersion, while the statistically significant W values represent modest, rather than complete, expert concordance.

### 3.4 Results of the expert consultation

Based on the experts' comments from the first Delphi round and in consideration of actual clinical practice, the research team conducted structured item review according to the statistical principles of the Delphi method. Experts agreed that the non-pharmacological labor pain management protocol for vaginal delivery should be structured around five major dimensions, including pain management objectives, pain management team building, pain assessment, pain management interventions, and outcome evaluation. For the secondary items, two items were introduced according to expert suggestions: shared decision-making for labor analgesia and maternal satisfaction with pain management. Experts also recommended categorizing non-pharmacological analgesia measures by mechanism of action. Referring to the 2023 Queensland Clinical Guidelines: Intrapartum Pain Management, the secondary items under “non-pharmacological pain management strategies” were classified into gate-control theory, central nervous system activation, and neurotransmitter release (5). Specific measures such as mobility and positioning, touch and massage, thermal therapy, breathing and relaxation, music therapy, and TENS were subsequently organized as tertiary items under the corresponding categories. The wording “individualized implementation of non-pharmacological labor analgesia measures” was used to emphasize individualized care. At the tertiary level, several items were introduced: “establish specific quality inspection standards for non-pharmacological labor analgesia management and conduct regular audits to ensure compliance”; “use a labor pain perception scale to evaluate maternal pain response and tolerance”; “provide antenatal information on available analgesic options, including the advantages and disadvantages of each”; “support the parturient's preferences and choices, collaboratively developing a labor pain management plan during the antenatal period, early labor, and between contractions”; “record pain scores immediately before and 30 min after analgesia interventions”; and “assess maternal satisfaction with pain management 1 day postpartum using a validated satisfaction questionnaire.” Tertiary items under the previous categories of mobility and positioning, touch and massage, thermal therapy, breathing and relaxation, music therapy, and TENS were integrated and reorganized according to intervention type. The wording “dynamically assess pain intensity according to labor progress or maternal condition changes” was used to strengthen the link between pain assessment and labor progression. Similarly, “implement non-pharmacological analgesia measures tailored to the woman's individual needs and labor progression” was used to emphasize person-centered implementation. In response to a safety concern regarding maternal self-regulation of the TENS device, the protocol specified that the midwife adjusts the device based on maternal feedback to achieve optimal comfort. Tertiary indicators focusing on epidural analgesia principles, general signs of labor, and partner access to healthcare facilities were not retained in the final protocol because they fell outside the defined scope of full-process non-pharmacological labor pain management.

Following first-round evaluation, the second-round questionnaire comprised five primary, 20 secondary, and 59 tertiary items.

During the second Delphi round, four written comments from three experts informed five predefined item-level decisions. Final terminology used “labor pain education”; training on pain knowledge and non-pharmacological labor analgesia methods was scheduled semiannually; and assessment of this knowledge and these methods was also scheduled semiannually. Two overlapping items were not retained because individualized implementation of non-pharmacological labor analgesia measures was already represented under D8 and shared decision-making concepts and communication skills were already represented under D7.

At protocol finalization, the framework consisted of five primary, 20 secondary, and 57 tertiary items (Table 1). The principal study finding is therefore a consensus-derived, full-process protocol that integrates organizational preparation, dynamic assessment, mechanism-organized supportive interventions, shared decision-making, and early postpartum outcome evaluation.

**Table 1:** Full-process non-pharmacological pain management protocol for vaginal delivery.

Item	Importance score (*x* ±*s*)	CV
A. Pain management objectives	4.91 ± 0.30	0.06
A1. Effective maternal coping with pain	4.91 ± 0.30	0.06
A1-1. Maintain pain within the mother's tolerable rang	5.00 ± 0.00	0.00
B. Pain management team building	5.00 ± 0.00	0.00
B1. Composition of the pain management team	5.00 ± 0.00	0.00
B1-1. The pain management team includes obstetricians, nurses, midwives, and doulas	4.82 ± 0.40	0.08
B1-2. Define specific responsibilities of each team member in pain management	4.91 ± 0.30	0.06
B2. Training and assessment of pain management team members	5.00 ± 0.00	0.00
B2-1. Conduct semiannual training on pain knowledge and non-pharmacological labor analgesia methods	4.73 ± 0.47	0.10
B2-2. Conduct semiannual assessments of pain knowledge and non-pharmacological labor analgesia methods	4.73 ± 0.47	0.10
B2-3. Emphasize the impact of healthcare providers' attitudes on maternal pain perception during training	4.18 ± 0.60	0.14
B3. Institutional framework	4.82 ± 0.40	0.08
B3-1. Establish institutional guidelines for full-process non-pharmacological labor analgesia management	4.82 ± 0.40	0.08
B3-2. Develop a standardized workflow for non-pharmacological labor analgesia management	4.91 ± 0.30	0.06
B3-3. Establish specific quality inspection standards for non-pharmacological labor analgesia management and conduct regular audits to ensure compliance	5.00 ± 0.00	0.00
C. Pain assessment	5.00 ± 0.00	0.00
C1. Timing and content of assessment	4.91 ± 0.30	0.06
C1-1. Upon admission, assess maternal knowledge and mastery of pain-coping strategies to identify acceptable methods	5.00 ± 0.00	0.00
C1-2. Assess pain site, nature, intensity, interval, duration, and related factors during labor onset, at 2.5 cm cervical dilation, at full dilation, and before analgesia	5.00 ± 0.00	0.00
C1-3. Assess the influence of emotional and psychological factors on maternal pain after labor onset	4.91 ± 0.30	0.06
C1-4. Dynamically assess pain intensity according to labor progress or maternal condition changes	5.00 ± 0.00	0.00
C1-5. Upon admission, assess maternal willingness to use non-pharmacological labor analgesia methods	4.91 ± 0.30	0.06
C2. Selection and application of assessment tools	5.00 ± 0.00	0.00
C2-1. Use the Visual Analog Scale (VAS) to assess pain intensity hourly during labor	4.91 ± 0.30	0.06
C2-2. Use a labor pain perception scale to evaluate maternal pain response and tolerance	4.91 ± 0.30	0.06
C2-3. Use a state anxiety inventory to assess maternal anxiety levels	4.45 ± 0.69	0.15
C3. Documentation of assessment results	4.91 ± 0.30	0.06
C3-1. Record pain site, nature, intensity, interval, duration, and related factors	5.00 ± 0.00	0.00
C3-2. Record pain scores, non-pharmacological analgesia methods, and implementation time	4.91 ± 0.30	0.06
C3-3. Reassess pain 30 min after non-pharmacological analgesia interventions	5.00 ± 0.00	0.00
D. Pain management interventions	5.00 ± 0.00	0.00
D1. Labor pain education	4.91 ± 0.30	0.06
D1-1. Courses cover normal and complicated labor and delivery, standard clinical nursing practices, available non-pharmacological pain management methods, and shared decision-making for labor analgesia	5.00 ± 0.00	0.00
D1-2. Instruction is delivered through individual or group sessions, online learning platforms, and video resources, guiding women and their families to master non-pharmacological labor analgesia methods prenatally	4.91 ± 0.30	0.06
D1-3. Develop and distribute a Manual for Non-Pharmacological Pain Management for Vaginal Delivery, including an overview of vaginal delivery, mechanisms and characteristics of labor pain, influencing factors, non-pharmacological analgesia measures, and the relationship between pain and delivery outcomes	4.91 ± 0.30	0.06
D1-4. Encourage the participation of the maternal partner in education sessions to provide information for supporting the parturient	4.64 ± 0.50	0.11
D1-5. Guide women in using pain assessment tools for self-evaluation of labor pain	5.00 ± 0.00	0.00
D2. Supportive environment	4.73 ± 0.47	0.10
D2-1. Ensure the birthing environment is comfortable, private, and quiet, allowing freedom of movement and easy access on and off the bed	4.91 ± 0.30	0.06
D2-2. Provide spaces for walking, bathing, and resting	4.73 ± 0.47	0.10
D3. Continuous labor support	4.73 ± 0.47	0.10
D3-1. Allow women to choose their labor companion, which may include partners, family members, friends, or doulas	4.73 ± 0.47	0.10
D3-2. Midwives and their team meet with the laboring woman and her companion before labor to understand their needs and expectations and establish a trusting relationship	4.91 ± 0.30	0.06
D3-3. Provide continuous intrapartum support, including guidance, reassurance, comforting touch, and assistance with positioning and movement, delivering ongoing non-medical care	4.91 ± 0.30	0.06
D3-4. Offer physical support such as holding hands or gentle touch, encourage and assist the woman with movement or position changes (e.g., squatting or standing), and provide basic care throughout labor	5.00 ± 0.00	0.00
D3-5. Provide emotional support by listening to concerns and using praise, reassurance, and encouragement to help the woman gain confidence and a sense of control	4.91 ± 0.30	0.06
D3-6. Offer informational support, providing details about labor progression, procedures, and available pain relief options to facilitate understanding and promote the use of non-pharmacological labor analgesia	5.00 ± 0.00	0.00
D4. Non-pharmacological pain management strategies—gate control theory	4.64 ± 0.67	0.14
D4-1. Apply sensory stimulation techniques to activate large-diameter nerve fibers, thereby inhibiting pain signal transmission	4.55 ± 0.52	0.11
D4-2. Activity and positioning: support women in adopting the most comfortable positions during labor and delivery; provide birthing balls for sitting, standing, or kneeling positions to promote trunk and pelvic relaxation; use peanut rollers to help maintain positions (e.g., placing a peanut roller under one leg in the lateral position)	5.00 ± 0.00	0.00
D4-3. Heat therapy: ensure the woman has intact sensory function before applying heat. Place 1–2 layers of cloth on the skin before heat application. For first-stage labor, apply warm packs to the back, waist, lower abdomen, and groin; for the second stage, apply heat to the perineum using hot water bags, warm compresses, or heated blankets. Temperature should be adjusted according to maternal comfort. Continuously monitor the skin and adjust duration or temperature as needed to prevent burns	4.91 ± 0.30	0.06
D4-4. Cold therapy: ensure intact sensory function before cold application. Place 1–2 layers of cloth between the skin and the cold source to avoid injury. During contractions, cold sources may be applied to the lower back to relieve lumbar and back pain when tolerated. Temperature should be adjusted according to maternal comfort. Discontinue cold therapy if shivering, marked discomfort, skin color change, or other intolerance occurs	4.36 ± 0.81	0.19
D4-5. Touch and massage: provided by midwives, nurses, or partners to establish tactile rapport during labor. Gentle tapping or stroking (starting at the feet) can be applied after labor onset. Praise the mother while performing touch or massage to promote relaxation during contractions and reduce pain and anxiety in the latent phase of the first stage	5.00 ± 0.00	0.00
D4-6. TENS: (1) When a woman elects TENS for pain relief, midwives provide support and guidance. (2) Electrodes are placed bilaterally at T10–L1 and S2–S4 levels. Continuous stimulation is applied during contractions, while pulsed mode is used during contraction intervals. The midwife adjusts the device based on maternal feedback to achieve optimal comfort. (3) TENS is contraindicated in women with pacemakers, sensory deficits, or during showering. When used with heat therapy, ensure heat devices are not applied directly over the electrodes	4.64 ± 0.67	0.14
D5. Non-pharmacological pain management strategies—SCNS activation	4.73 ± 0.47	0.10
D5-1. Use cognitive and psychological training to stimulate the amygdala and limbic system, triggering endogenous analgesic responses	4.64 ± 0.50	0.11
D5-2. Breathing and relaxation: during the latent phase of the first stage of labor, encourage the woman and her partner to perform rhythmic breathing exercises during contraction intervals. Slow rhythmic breathing (6–12 breaths/min) and slightly faster paced breathing (30–60 breaths/min) are recommended to reduce labor pain and anxiety	4.91 ± 0.30	0.06
D5-3. Provide additional support, such as mindfulness therapy, when significant labor-related anxiety is identified	4.91 ± 0.30	0.06
D6. Non-pharmacological pain management strategies—neurotransmitter release	4.64 ± 0.50	0.11
D6-1. Modulate neuronal activity and potentially engage endogenous analgesic processes, such as endorphin release, which may contribute to enhanced pain tolerance	4.73 ± 0.47	0.10
D6-2. Music therapy: before labor, communicate with the woman to understand her music preferences. Select music or ambient sounds that positively influence mood and match labor stages. Play the chosen music during labor to promote relaxation and improve emotional state	5.00 ± 0.00	0.00
D6-3. Acupressure: during the first stage of labor, apply manual or fixed vaccaria seed (*Semen Vaccariae*) pressure to *Sanyinjiao* (SP6) and *Hegu* (LI4) acupoints. Pressure should be applied to the point of mild soreness, distension, or radiating sensation. The midwife performs sequential acupressure on these points once every 15 min, with each session lasting 1 min	4.91 ± 0.30	0.06
D7. Shared decision-making for labor analgesia	4.91 ± 0.30	0.06
D7-1. Provide antenatal information on available analgesic options, including the advantages and disadvantages of each	4.91 ± 0.30	0.06
D7-2. Support the parturient's preferences and choices, collaboratively developing a labor pain management plan during the antenatal period, early labor, and between contractions	5.00 ± 0.00	0.00
D8. Individualized implementation of non-pharmacological labor analgesia measures	5.00 ± 0.00	0.00
D8-1. Encourage the woman to express her pain relief needs at any point during labor	4.82 ± 0.60	0.13
D8-2. Implement non-pharmacological analgesia measures tailored to the woman's individual needs and labor progression	5.00 ± 0.00	0.00
D8-3. Reassess pain 30 min after each intervention	4.91 ± 0.30	0.06
E. Pain management outcome evaluation	5.00 ± 0.00	0.00
E1. Pain scores	4.91 ± 0.30	0.06
E1-1. Record pain scores during labor, at 2.5 cm cervical dilation, and at full dilation	4.91 ± 0.30	0.06
E1-2. Record pain scores immediately before and 30 min after analgesia interventions	4.91 ± 0.30	0.06
E1-3. Assess labor pain perception within 2 h postpartum using a validated pain perception scale	4.91 ± 0.30	0.06
E2. Sense of control during labor	4.64 ± 0.50	0.11
E2-1. Evaluate maternal sense of control 1 day postpartum using the Labor Agentry Scale	4.82 ± 0.40	0.08
E3. Anxiety	4.36 ± 0.67	0.15
E3-1. Assess maternal anxiety at 37 weeks of gestation, during labor, and within 2 h postpartum	4.55 ± 0.52	0.11
E4. Labor outcomes	5.00 ± 0.00	0.00
E4-1. Document the duration of the first and second stages of labor, delivery mode, postpartum hemorrhage within 2 h, and neonatal Apgar scores	5.00 ± 0.00	0.00
E5. Maternal satisfaction with pain management	4.91 ± 0.30	0.06
E5-1. Assess maternal satisfaction with pain management 1 day postpartum using a validated satisfaction questionnaire	4.91 ± 0.30	0.06

CV values were calculated from unrounded source data and are presented to two decimals. D4-4 (cold therapy) had the highest CV (0.19) and is retained as a conditional item requiring individualized assessment, careful application, and prospective clinical validation.

## 4 Discussion

### 4.1 Necessity and significance of a non-pharmacological labor pain management protocol for women undergoing vaginal delivery

Women undergoing vaginal delivery require continuous, individualized support for labor pain because pain perception is shaped by labor progress, previous experience, emotional state, social support, and care environment. Persistent labor pain may contribute to negative birth experience and reduced sense of control. Non-pharmacological labor analgesia can help women cope with pain, support maternal participation, and complement pharmacological analgesia, but its effect varies by technique, timing, and implementation context (1, 2). In China, the implementation of non-pharmacological pain management services remains insufficiently standardized. Domestic literature and midwifery practice indicate that some non-pharmacological interventions are complex, inconsistently assessed, or insufficiently tailored to women's needs (7). Additionally, many studies focus on specific labor stages. For example, Huang et al. applied evidence-based nursing methods to identify non-pharmacological pain management strategies but implemented these strategies mainly during the latent phase of labor (20). Zhang et al. provided one-on-one doula support combined with non-pharmacological labor analgesia from 3 cm cervical dilation to the postpartum period, using interventions such as birthing balls, music therapy, freedom of movement, and paced breathing (21). Internationally, Gallo et al. applied sequential non-pharmacological interventions during labor, implementing birthing ball exercises, aromatherapy, and other analgesia measures at 4–5 cm, 5–6 cm, and >7 cm cervical dilation (6). However, these models provide limited guidance for organizing the entire process from prenatal preparation to early postpartum evaluation. After surveying labor services in several midwifery institutions in Shanghai, Zhu et al. recommended providing individualized, continuous comfort measures and supportive services throughout labor (22). Accordingly, this study used the four-stage framework of the Manchester pain management model to construct a full-process non-pharmacological labor pain management protocol for women undergoing vaginal delivery, with the aim of supporting standardization and providing a basis for future clinical validation.

### 4.2 Specialty and targeted nature of the full-process non-pharmacological labor pain management protocol for women undergoing vaginal delivery

Women undergoing vaginal delivery require continuous, individualized support for labor pain, and inadequate pain management can affect cooperation, psychological comfort, and perceived birth experience. Compared with isolated non-pharmacological labor analgesia techniques, the proposed protocol offers greater standardization, integration, and individualization. The protocol is structured around five domains, namely pain management objectives, pain management team building, pain assessment, pain management interventions, and outcome evaluation, thereby organizing the full-process management of non-pharmacological labor analgesia for women undergoing vaginal delivery. In defining the pain management objectives, the protocol establishes criteria for effective labor pain control, helping women cope with labor discomfort within a tolerable range. The pain management team comprises obstetricians, ward nurses, midwives, and doulas, with active participation from the woman and her family in shared decision-making. The protocol is intended for obstetric units providing vaginal delivery services across primary, secondary, and tertiary care settings. In tertiary centers where neuraxial labor analgesia is routinely available, it is intended to complement pharmacological analgesia and support shared decision-making; in primary or secondary facilities where pharmacological options may be limited, it provides an implementation-oriented framework for standardized, low-risk supportive care. Multi-disciplinary collaboration supports continuous maternal care throughout childbirth, while institutional workflows and quality control standards provide a basis for future implementation and evaluation.

This protocol differs from existing domestic and international studies in two core aspects: continuity of intervention timing and systematic integration of interventions. Regarding intervention timing, previous studies and protocols often focus on selected techniques or specific stages, such as latent-phase interventions, TENS, or birthing ball exercises (20, 23, 24), whereas the present protocol extends non-pharmacological analgesia across the full continuum from prenatal education through intrapartum interventions to postpartum outcome evaluation within 24 h. This addresses the limitations of fragmented interventions in preceding research. For example, the inclusion of postpartum pain assessment items, such as the labor pain perception scale within 2 h postpartum, responds to the need for early recovery-stage evaluation in full-process labor management. International sequential intervention protocols, though spanning multiple labor stages, typically focus on physical interventions and provide less detail on structured elements such as pain management team building, standardized assessment, shared decision-making, and outcome evaluation (5, 6, 25, 26). In contrast, this protocol integrates team training and assessment, dynamic evaluation mechanisms, and individualized intervention adjustment, forming a closed-loop system structured around objectives, team building, assessment, intervention, and evaluation.

Through its structured design, the protocol may support continuous tracking and optimization of labor pain management, but its clinical effectiveness still requires prospective validation. During the prenatal education phase, instructing women to use pain assessment tools (D1-5) may enhance their pain-coping ability and support active participation in pain management. In the intrapartum phase, interventions are dynamically adjusted according to cervical dilation and maternal needs, for instance, reinforcing breathing and relaxation training at 2.5 cm dilation (D5-2) and adding perineal heat therapy during the second stage (D4-3), combined with hourly pain assessments (C2-1) and re-evaluation 30 min post-intervention (C3-3) to align interventions with real-time changes in pain intensity. During the postpartum phase, pain scores at 2 h (E1-3) and satisfaction assessments at 1 day (E5-1) create a closed-loop feedback system that can inform ongoing protocol optimization. Expert consultation results support the acceptability of this full-process design. Among the 57 tertiary items covering prenatal preparation to 24 h postpartum, mean importance scores ranged from 4.18 to 5.00, with CV values of 0.00–0.19. Items reflecting full-process continuity, such as dynamic pain intensity assessment (C1-4) and individualized implementation of analgesic measures (D8-2), achieved mean scores of 5.00 and CV values of 0.00, indicating unanimous expert recognition of their importance.

### 4.3 Scientific basis and reliability of the full-process non-pharmacological labor pain management protocol for women undergoing vaginal delivery based on the Manchester pain management model

The Manchester pain management model emphasizes recognizing individual differences in pain perception and considering factors that influence pain management, thereby enabling systematic pain treatment and nursing care. The primary domains of this protocol are derived from the four stages of the model, i.e., preparation, assessment, intervention, and recovery. Specifically, the preparation stage addresses the establishment of pain management objectives and the construction of the pain management team; the assessment stage focuses on pain scoring and evaluation of influencing factors; the intervention stage involves implementation of pain management measures; and the recovery stage emphasizes evaluation of analgesic outcomes.

Furthermore, this study conducted a structured evidence review to analyze domestic and international evidence on non-pharmacological labor analgesia for vaginal delivery, extracting relevant findings and combining them with clinical practice experience to develop the preliminary protocol. This approach supports the evidence-informed foundation of the protocol. Subsequently, two rounds of Delphi expert consultation were conducted to refine and finalize the protocol. The 11 consulted experts were drawn from tertiary hospitals across multiple provinces/cities, with extensive clinical experience and research competence. Their professional expertise spanned obstetrics, ward nursing, midwifery, nursing management, and pain management. Both rounds of questionnaires were fully returned, achieving a 100% response rate and indicating high expert engagement. The Cr value across both rounds was 0.92, reflecting acceptable expert authority. Kendall's W values across item levels ranged from 0.208 to 0.240 and were statistically significant, demonstrating modest but improving concordance among experts. These findings indicate that the developed full-process non-pharmacological labor pain management protocol for vaginal delivery has an acceptable consensus basis for subsequent implementation research.

This protocol adheres to the four-stage logic of the Manchester pain management model, with explicit mapping of each stage to specific items. The preparation stage corresponds to pain management objectives (A) and pain management team building (B), establishing the foundation for full-process interventions through measures such as setting pain tolerance standards, forming a multidisciplinary team, and developing quality control procedures (B3-3). The assessment stage is represented by pain assessment (C), which includes dynamic evaluations during labor at cervical dilation of 2.5 cm and full dilation (C1-2) and hourly monitoring using the VAS (C2-1), enabling real-time tracking of pain intensity and psychological needs. The intervention stage encompasses pain management interventions (D), including touch and massage based on the gate control theory (D4-5) and acupressure categorized within the neurotransmitter-release domain (D6-3). These mechanism-based categories provide an organizational framework, while the specific biological pathways remain incompletely established. Individualized implementation (D8) ensures that interventions are synchronized with labor progression. The recovery stage is reflected in pain management outcome evaluation (E), including postpartum pain scoring at 2 hours (E1-3) and satisfaction assessment at 1 day (E5-1), forming a closed-loop feedback system for intervention evaluation. This mapping is summarized in Supplementary Table 1, and the design complements prior evidence on non-pharmacological labor pain management by organizing discrete interventions into a staged protocol (27).

### 4.4 Obstetric specialty features of the protocol

The obstetric specialty of this protocol is reflected in its adaptation to the labor and delivery context. Compared with pain management in other clinical settings (28), its distinct features include labor stage-specific interventions, maternal and neonatal safety orientation, and family involvement. In terms of labor stage-specific interventions, measures are tailored to different stages of cervical dilation, for example, emphasizing breathing and relaxation during the first stage and enhancing perineal heat therapy during the second stage, differing from fixed-frequency postoperative analgesic regimens in surgical settings. For maternal and neonatal safety orientation, interventions specify contraindications and precautions, such as avoiding TENS in women with pacemakers and protecting the skin during heat or cold therapy. Regarding family involvement, items such as spousal touch and labor companionship incorporate family members into supportive care, aligning with family-centered obstetric practice. These features indicate that the protocol is a clinical practice and implementation protocol rather than an assessment tool; the assessment items embedded in domains C and E are designed to guide clinical decision-making and outcome monitoring within the protocol.

### 4.5 Limitations

This study has several limitations. Although the protocol was developed through structured evidence review and expert consultation, it has not yet been tested in clinical practice to determine its actual impact on outcomes such as pain scores, cesarean delivery rates, maternal satisfaction, or neonatal outcomes. As a result, discrepancies may exist between protocol design and practical implementation; for instance, semiannual training and assessment of staff may be more challenging to execute in primary hospitals than anticipated. The consulted experts were primarily from eastern coastal regions, so applicability in medical institutions in central and western China requires further validation. The Delphi panel included clinical and management experts but did not include women with childbirth experience or patient representatives, which may have limited direct incorporation of service-user preferences into item prioritization. Additionally, some items exhibited relatively high variability in the second round of consultation, reflecting areas of clinical controversy. For example, the cold therapy item (D4-4) had a CV of 0.19, mainly due to differing opinions regarding its suitability: some obstetricians expressed concerns that individual tolerance to cold during contractions varies, potentially causing shivering or discomfort, and recommended restricting its use; on the other hand, midwifery experts highlighted its immediate effect in alleviating back pain and advocated for retention with detailed operational guidance. Similarly, the state anxiety assessment (C2-3) had a CV of 0.15, reflecting differing views on the relevance of psychological evaluation to pain management. Nursing managers emphasized the amplifying effect of anxiety on pain perception, while clinicians focused primarily on pain itself, indicating the need for flexible adjustment of assessment dimensions based on institutional context. Future studies should conduct multicenter clinical trials to evaluate the protocol's feasibility, acceptability, and effectiveness and should incorporate regional adaptations and maternal perspectives to enhance its generalizability. Therefore, the protocol should be interpreted as a consensus-based implementation framework rather than evidence of demonstrated clinical benefit.

## 5 Conclusion

Guided by the Manchester pain management model, this Delphi-based protocol development study translated structured evidence and multidisciplinary expert consensus into a full-process non-pharmacological labor pain management protocol for women undergoing vaginal delivery. The key findings were a 100% response rate in both consultation rounds, an expert authority coefficient of 0.92, final item-level CV values of 0.00–0.19, and statistically significant but modest concordance across item levels (Kendall's W = 0.212–0.240), culminating in five primary domains, 20 secondary items, and 57 tertiary items. The study therefore shows that fragmented evidence on individual techniques can be organized into a standardized pathway spanning prenatal preparation, intrapartum support, and outcome evaluation through 24 h postpartum. The protocol is intended to complement pharmacological analgesia and support consistent, individualized care; its feasibility, acceptability, and clinical effects should be evaluated in future multicenter prospective studies.

## Data Availability

The original contributions presented in the study are included in the article/Supplementary material, further inquiries can be directed to the corresponding author.
