# Supplementary material for: Development of a full-process non-pharmacological labor pain management protocol for vaginal delivery: a Delphi study
Source: Front Med (Lausanne). 2026 Oct 7;13:1849246. doi: 10.3389/fmed.2026.1849246 (PMC13683653; doi:10.3389/fmed.2026.1849246)
Supplement: Supplementary file 1 [file Supplementary_file_1.docx]

**Supplementary Table 1. Mapping of the final protocol to the Manchester pain management model**

| **Manchester model stage** | **Corresponding protocol domain** | **Representative final items** | **Function in the full-process protocol** |
| --- | --- | --- | --- |
| Preparation | Pain management objectives (A) and pain management team building (B) | A1, B1, B2, B3 | Defines tolerable pain-management goals, establishes a multidisciplinary team, clarifies responsibilities, and sets training, assessment, workflow, and quality-control requirements before implementation. |
| Assessment | Pain assessment (C) | C1, C2, C3 | Guides assessment of pain site, nature, intensity, duration, related factors, anxiety, willingness to use non-pharmacological methods, documentation, and reassessment after interventions. |
| Intervention | Pain management interventions (D) | D1–D8 | Organizes labor pain education, supportive environment, continuous labor support, mechanism-based non-pharmacological strategies, shared decision-making, and individualized implementation according to labor progress and maternal needs. |
| Recovery and evaluation | Pain management outcome evaluation (E) | E1–E5 | Evaluates pain scores, sense of control, anxiety, labor outcomes, and maternal satisfaction within the early postpartum period to support closed-loop protocol optimization. |
